# The cinnamyl alcohol dehydrogenase gene family in *Populus*: phylogeny, organization, and expression

**DOI:** 10.1186/1471-2229-9-26

**Published:** 2009-03-06

**Authors:** Abdelali Barakat, Agnieszka Bagniewska-Zadworna, Alex Choi, Urmila Plakkat, Denis S DiLoreto, Priyadarshini Yellanki, John E Carlson

**Affiliations:** 1 The School of Forest Resources, The Huck Institutes of the Life Sciences, Pennsylvania State University, 324 Forest Resources Building, University Park, PA 16802, USA; 2 Department of General Botany, Institute of Experimental Biology, Adam Mickiewicz University, Umultowska 89, 61-614 Poznań, Poland; 3 Schreyer Honors College, Pennsylvania State University, 10 Schreyer Honors College, University Park, PA 16802, USA; 4 The School of Forest Resources, Department of Horticulture, The Huck Institutes of the Life Sciences, Pennsylvania State University, 323 Forest Resources Building, University Park, PA 16802, USA

## Abstract

**Background:**

Lignin is a phenolic heteropolymer in secondary cell walls that plays a major role in the development of plants and their defense against pathogens. The biosynthesis of monolignols, which represent the main component of lignin involves many enzymes. The cinnamyl alcohol dehydrogenase (CAD) is a key enzyme in lignin biosynthesis as it catalyzes the final step in the synthesis of monolignols. The CAD gene family has been studied in *Arabidopsis thaliana, Oryza sativa *and partially in *Populus*. This is the first comprehensive study on the CAD gene family in woody plants including genome organization, gene structure, phylogeny across land plant lineages, and expression profiling in *Populus*.

**Results:**

The phylogenetic analyses showed that CAD genes fall into three main classes (clades), one of which is represented by CAD sequences from gymnosperms and angiosperms. The other two clades are represented by sequences only from angiosperms. All *Populus *CAD genes, except *PoptrCAD 4 *are distributed in Class II and Class III. CAD genes associated with xylem development (*PoptrCAD 4 and PoptrCAD 10*) belong to Class I and Class II. Most of the CAD genes are physically distributed on duplicated blocks and are still in conserved locations on the homeologous duplicated blocks. Promoter analysis of CAD genes revealed several motifs involved in gene expression modulation under various biological and physiological processes. The CAD genes showed different expression patterns in poplar with only two genes preferentially expressed in xylem tissues during lignin biosynthesis.

**Conclusion:**

The phylogeny of CAD genes suggests that the radiation of this gene family may have occurred in the early ancestry of angiosperms. Gene distribution on the chromosomes of *Populus *showed that both large scale and tandem duplications contributed significantly to the CAD gene family expansion. The duplication of several CAD genes seems to be associated with a genome duplication event that happened in the ancestor of *Salicaceae*. Phylogenetic analyses associated with expression profiling and results from previous studies suggest that CAD genes involved in wood development belong to Class I and Class II. The other CAD genes from Class II and Class III may function in plant tissues under biotic stresses. The conservation of most duplicated CAD genes, the differential distribution of motifs in their promoter regions, and the divergence of their expression profiles in various tissues of *Populus *plants indicate that genes in the CAD family have evolved tissue-specialized expression profiles and may have divergent functions.

## Background

Lignin is a phenolic heteropolymer that provides plant cells with structural rigidity, a barrier against insects and other pestilent species, and hydrophobicity [1-4]. Its role in hydrophobicity helps xylem cells facilitate the conduction of water and minerals throughout the plant [5]. Lignin is the second most abundant plant molecule on earth next to cellulose and comprises approximately 35% of the dry matter of wood in some tree species [6]. The composition of lignin consists of various phenylpropanoids, predominantly the monolignols *p*-coumaryl, coniferyl, and sinapyl alcohols. Lignin varies in content and composition between gymnosperms and angiosperms. In gymnosperms, lignin contains guaiacyl subunits (G units) and *p*-hydroxyphenyl units (H units) polymerized from coniferyl alcohol and from *p*-coumaryl alcohol respectively. Lignin in angiosperms comprises, in addition to G-units and some H-units [7], syringyl units (or S-units) polymerized from sinapyl alcohol. However, there are exceptions found within each group [7] and variation in lignin composition can even occur between cell types within the same plant.

The monolignol biosynthetic pathway involves many intermediates and enzymes [8]. The first step in the process consists of a deamination of phenylalanine by the phenylalanine ammonia-lyase (PAL) [9,10] that produces cinnamic acid. Cinnamic acid is then hydroxylated by the enzyme cinnamate-4-hydroxylase (C4H) producing *p*-coumaric acid [11], which is in turn activated by 4-coumarate:CoA ligase (4CL) to produce *p*-coumaroyl-CoA [12,13]. This product is processed by cinnamoyl-CoA reductase (CCR) to coniferaldehyde, which in turn is converted to coniferyl alcohol by the action of CAD. *p*-coumaroyl-CoA can also be transformed to *p*-coumaroyl-CoA shikimate by the action of hydroxycinamoyl transferase (HCT). *p*-coumaroyl-CoA shikimate proceeds through a series of transformations into caffeoyl shikimate, caffeoyl-CoA, feruloyl CoA, and coniferaldehyde by the action of the enzymes *p*-coumarate 3-hydrolase (C3H), HCT, caffeoyl-CoA O-methyltransferase (CCOMT), and cinnamoyl CoA reductase (CCR), respectively. Coniferaldehyde can be transformed to coniferyl alcohol by the action of CAD or lead to 5-Hydroxy- coniferaldehyde and sinapyl aldehyde under the action of ferulate 5-hydrolase (F5H) and caffeic/5-hydroxyferulic acid O-methyltransferase (COMT). The sinapyl alcohol is produced either from sinapyl aldehyde by CAD or from coniferyl alcohol by F5H and COMT. It has also been reported that the synthesis of sinapyl alcohol can be catalyzed by sinapyl alcohol dehydrogenase (SAD) [14]. However, recent studies [15,16] did not find any detectable sinapyl alcohol dehydrogenase activity in *Arabidopsis *and *Oryza *indicating that the same CAD gene products can synthesize both coniferyl and sinapyl alcohols.

Because of its economic importance and biological role in various developmental and defense processes, the function of lignin biosynthesis related genes has been well studied in various plants [17,18]. Down-regulation of genes involved in the early steps of the monolignol synthesis pathway can lead to a reduction in lignin biosynthesis [17]. However, altered expression of CAD genes in various plants resulted in only slight variations in lignin content [19-23]. This is mainly due to the incorporation of other phenolic products that compensate for monolignols in lignin as well as the compensation by other members of the CAD gene family. A significant reduction of lignin was detected in natural CAD mutants in *Pinus *(5%) and the *bm2*, *bm3*, and *bm4 *mutants in maize (20%) [24,25]. The gene underlying the *bm1 *mutant in maize is not a CAD gene, however, and may encode a regulator of several CAD genes. Down-regulating the expression of CAD genes in *Nicotiana tabacum*, *Populus*, and *Pinus *showed no gross morphological variations but CAD deficient plants were enriched in coniferyl aldehyde and sinapyl aldehyde [24,26,27]. The accumulation of the aldehyde molecules is responsible for the red-brown color in the stems of natural and induced CAD mutants in *Populus*, *Zea*, *Oryza*, and *Pinus *[15,16,24,25]. A recent study in *Arabidopsis *showed that double mutants in the two major CAD genes associated with lignin biosynthesis (*AtCAD_C *and *AtCAD_D *named *AtCAD4 *and *AtCAD5*) present prostrate stems because of the weakness of the vasculature [15]. A reduction in the size and the diameter of the stems was also observed in the double mutant plants. Beside its role in plant development, CAD also seems to play a key role in plant defense against abiotic and biotic stresses [1,28,29].

CAD proteins are encoded by a gene family in plants [29,30]. Complete sets of *CAD *genes and *CAD-like *genes have been previously identified in the genomes of model species (*Arabidopsis*, *Oryza*, and *Populus*) and partially from expressed sequences of non-model plants. In *Arabidopsis*, CAD exists as a multigene family consisting of nine genes (*AtCAD1 *to *AtCAD9*) [31,32]. Although all nine have been classified as CAD genes based on their predicted protein sequences, only *CAD-C *(*AtCAD5*) and *CAD-D *(*AtCAD4*) have been shown to have major roles in lignin synthesis in *Arabidopsis *[32,33]. *AtCAD7 *and *AtCAD8 *may also be involved to some extent in lignin biosynthesis [33]. *AtCAD2*, *AtCAD3*, *AtCAD6*, and *AtCAD9 *appear to encode mannitol dehydrogenases. A double mutation of *AtCAD2 *and *AtCAD6 *led to an over-expression of *AtCAD1 *(*AtCAD7*) suggesting a compensation between some CAD genes [34]. In *Oryza*, 12 CAD genes have been reported [16].

Phylogenetic analysis [29,35] of the predicted amino acid sequences of CAD genes in *Arabidopsis *has shown that CAD is organized into three classes with gymnosperm sequences clustering in a separate group [29]. On the contrary, another study [30] showed that CAD genes were distributed in two classes both containing monocot and eudicot genes. The contradictory results obtained in these two studies were obtained using a limited set of genes and were not conclusive.

In this study we retrieved and compared CAD sequences from a wide variety of plants, making full use of the available plant genome sequences (*Arabidopsis*, *Oryza*, *Populus*, *Medicago*, and *Vitis*) as well as expressed sequence databases for species of basal angiosperms, gymnosperms, and mosses. This dataset was used to analyze the phylogeny of the CAD gene family. We also analyzed the organization, the structure, and the expression of CAD genes in *Populus*. This provided insight into the evolution of their structure and function as well as mechanisms that contributed to gene duplications.

## Results

### CAD gene family organization

In model species for which the genome is completely sequenced, 71 CAD genes have been identified to date (see Additional file 1): 9 in *Arabidopsis *[36], 12 in *Oryza *[30], 15 in *Populus *(this study), 18 in *Vitis *(this study), and 17 in *Medicago *(this study). Furthermore, we identified 54 more CAD genes in 31 other species, which include a variety of eudicots, monocots, basal angiosperms, and gymnosperms. Additional file 1 includes the list of these CAD gene names based on the standard established by the International *Populus *Genome Consortium (IPGC)[35] with the names of species (Poptr for *Populus trichocarpa *for example), the protein name (CAD), and a designation of family and clade memberships derived from this study. Additional file 1 also provides the accession number and database source for each gene.

**Figure 1:**
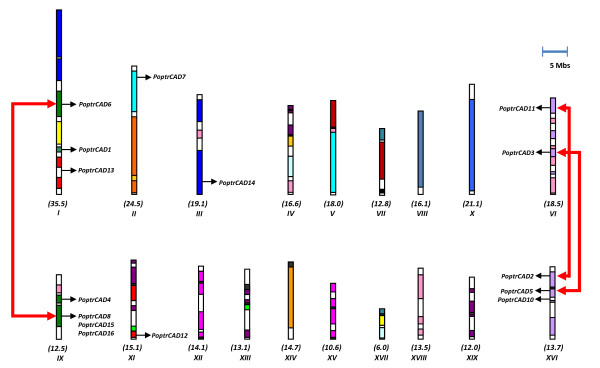
**Distribution of CAD genes on *Populus *chromosomes**. The names of the chromosomes and their sizes (Mb) are indicated below each chromosome. Segmental duplicated homeologous blocks [39] are indicated with the same color. The position of genes is indicated with an arrowhead.

Analysis of the physical gene distribution in the *Arabidopsis *and *Populus *genomes showed that most CAD genes were located on duplicated blocks. In *Arabidopsis *only one gene (*AtCAD5*) is not located on duplicated chromosomal blocks. Almost all of the genes are still in conserved positions within the duplicated blocks. In *Populus*, we found 14 of the 15 CAD genes distributed on duplicated regions. The *Populus *CAD genes were distributed on seven chromosomes with chromosomes I, IX, and XVI having three or more genes each (Fig. 1). *PoptrCAD9 *was located on a scaffold not yet assigned to a chromosome (see Additional file 1). Homologous pairs from the nine duplicated genes (*PoptrCAD6*, *PoptrCAD11*, *PoptrCAD3*, *PoptrCAD4*, *PoptrCAD15*, *PoptrCAD16*, *PoptrCAD8*, *PoptrCAD2*, and *PoptrCAD5*) remain in conserved positions on homeologous duplicated blocks. Duplicates of *PoptrCAD1*, *PoptrCAD12*, *PoptrCAD7*, and *PoptrCAD14 *appear to be lost from the *Populus *genome by an unknown gene death mechanism. *PoptrCAD8*, *PoptrCAD16*, and *PoptrCAD15 *seem to be generated via tandem duplications from one of the genes. Only *PoptrCAD13 *and *PoptrCAD10 *were not located on duplicated blocks.

In *Oryza *five CAD genes (*OsCAD2*, *OsCAD9*, *OsCAD10*, *OsCAD11*, and *OsCAD8*) were located on duplicated segments. Four CAD genes in rice (*OsCAD8A*, *OsCAD8B*, *OsCAD8C*, and *OsCAD8D*) were distributed one after the other at the same locus [30] indicating a possible tandem duplication origin.

### Intron-exon structure of CAD genes

**Figure 2:**
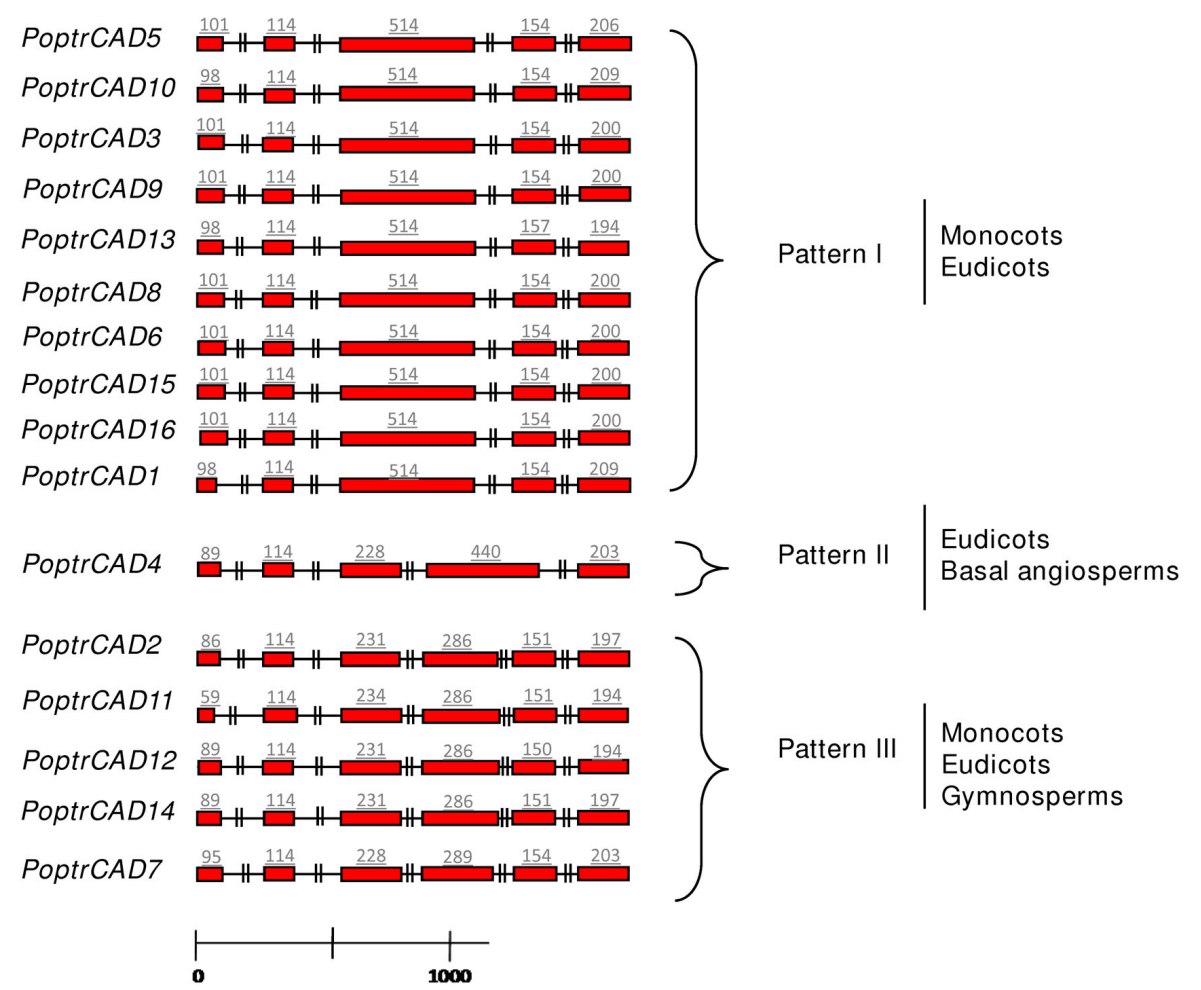
**Intron-exon structures of CAD genes from *Populus***. Exons and introns are indicated by open boxes and lines respectively. Numbers above boxes indicate the exon sizes. The intron sizes are not to scale. The names of CAD genes and intron-exon structure are indicated at the left and right sides respectively.

Gene structure analysis of *Populus *CAD genes (Fig. 2) revealed the existence of three patterns of intron-exon structures. Pattern 1 (*PoptrCAD5*, *PoptrCAD10*, *PoptrCAD3*, *PoptrCAD9*, *PoptrCAD1*, *PoptrCAD13*, *PoptrCAD8*, *PoptrCAD6*, *PoptrCAD15*, and *PoptrCAD16*), pattern 2 (*PoptrCAD4*), and pattern 3 (*PoptrCAD2*, *PoptrCAD11*, *PoptrCAD12*, *PoptrCAD14*, and *PoptrCAD7*) were composed by 5, 5, and 6 exons, respectively. Pattern 1 and pattern 2 present a difference in length of exon 3 and exon 4. Genes within these patterns present a similar number and size of exons. All *Populus *duplicated genes show a similar structure. *PoptrCAD16 *and *PoptrCAD8*, which may have risen from *PoptrCAD15 *by tandem duplication, also showed the same structure. While the intron length is conserved between some homeologous introns, others exhibit a great deal of variation. The increase in length could be due to transposable element insertions. Homeologous duplicate pairs (*PoptrCAD11 *– *PoptrCAD2*, *PoptrCAD5 – PoptrCAD3*, and *PoptrCAD6 *– *PoptrCAD8*) genes also show similar structure between homologs (Fig. 2).

The number of different intron/exon patterns for *Populus *(this study), *Oryza *[30], and *Arabidopsis *[31] totaled three, four, and six, respectively. Pattern 1 and pattern 3 of intron-exon structure were common to eudicots and monocots, while pattern 2 was found only in eudicots. It is important to note that *Oryza *has the greatest number of intron-exon structure variants even though rice has fewer CAD genes than *Populus *and apparently less overall chromosomal duplications.

### Promoter sequence analysis

Analysis of promoter sequences of the *Populus *CAD genes allowed us to identify several motifs that are known to be involved in the regulation of gene expression in various developmental and physiological processes (Table 1 and see Additional file 2). Some of those motifs interact with known regulators of genes involved in lignin biosynthesis such as *Myb *and *Zinc finger *genes [37]. The other motifs are involved in the response to various hormones involved in responses to biotic and abiotic stresses such as auxin, ethylene, abscisic acid (ABA), salicylic acid, and Methyl Jasmonate (MeJA) (Brill et al., 1999; Mur et al., 1996; Yasuda et al., 2008; Lawrence et al., 2006). *PoptrCAD4 *and *PoptrCAD10*, which are both preferentially expressed in xylem, possess transcription factor binding motifs involved in development and in response to various stresses, but showed some differences in their sets of motifs and in the distribution of the motifs in their promoter regions. For instance, *PoptrCAD4 *has motifs involved in response to ABA, stress, MeJA, wounding, and light. Unlike *PoptrCAD4*, *PoptrCAD10 *has motifs that bind to Myb and zinc finger proteins or are involved in response to auxin. Some CAD genes such as *PoptrCAD1*, *PoptrCAD2*, *PoptrCAD10*, and *PoptrCAD11 *possess promoter motifs involved in the response to fungal elicitors. Other genes (*PoptrCAD2*, *PoptrCAD4*, *PoptrCAD5*, *PoptrCAD7*, *PoptrCAD9*, *PoptrCAD10*, *PoptrCAD16*) possess motifs involved in response to wounding, herbivore stress, as well as other stresses.

**Table 1:** List of motifs found in the promoter regions of *Populus *CAD genes.

	Salicylic acid	Auxin	Defense/stress responsiveness	Fungal elicitor	Methyl-jasmonate	Myb binding	Wound	Transcription Enhancer	Zinc finger binding	Ethylene	Herbivore defense	Abscisic Acid	Light responsiveness
*PoptrCAD1*	**X**			**X**						**X**			**X**
*PoptrCAD2*	**X**			**X**	**X**		**X**						
*PoptrCAD3*		**X**	**X**		**X**	**X**		**X**	**X**				**X**
*PoptrCAD4*	**X**		**X**		**X**		**X**					**X**	**X**
*PoptrCAD5*	**X**		**X**		**X**		**X**						
*PoptrCAD6*		**X**							**X**	**X**			**X**
*PoptrCAD7*	**X**	**X**		**X**	**X**	**X**	**X**	**X**					**X**
*PoptrCAD8*	**X**	**X**			**X**	**X**					**X**	**X**	**X**
*PoptrCAD9*	**X**	**X**					**X**	**X**	**X**				**X**
*PoptrCAD10*	**X**	**X**							**X**				
*PoptrCAD11*				**X**	**X**		**X**			**X**		**X**	
*PoptrCAD12*					**X**	**X**						**X**	
*PoptrCAD13*	**X**										**X**		
*PoptrCAD14*					**X**			**X**					**X**
*PoptrCAD15*			**X**										**X**
*PoptrCAD16*	**X**						**X**		**X**			**X**	**X**

### Evolution of CAD genes

**Figure 3:**
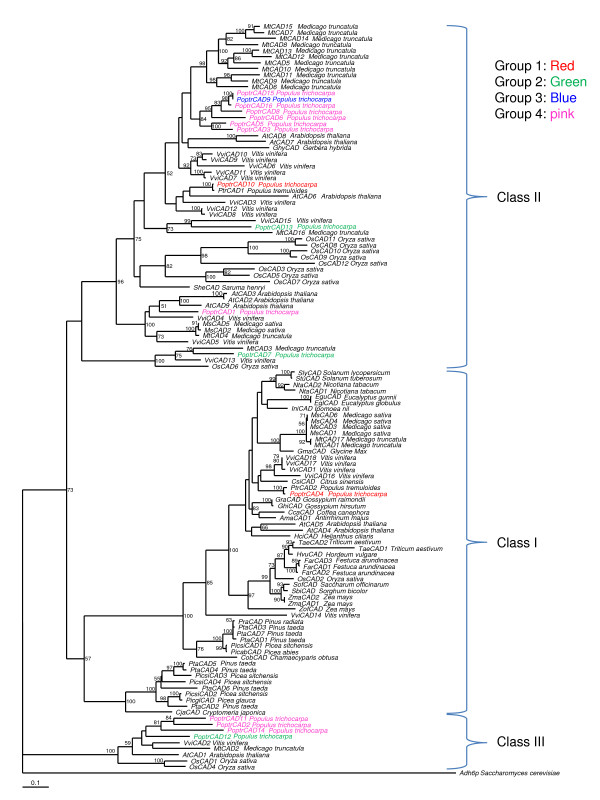
**Maximum Likelihood bootstrap tree phylogeny based on amino acid sequences of CAD genes in various land plants**. Numbers above branches refer to NJ bootstrap values. Brackets highlight the three classes of CAD genes. Colors indicate gene groups determined based on their expression in various *Populus *plant tissues. Red (group 1), green (group 2), and blue (group 3) indicate genes preferentially expressed in xylem, leaves, as well as leaves and xylem respectively. Pink (group 4) represents genes that showed no preferential expression between *Populus *tissues.

Maximum Likelihood (ML) bootstrap trees (based on nt and AA alignments) indicate that the CAD genes of land plants consist of three classes (Fig. 3). The distribution of these three classes was supported by relatively high bootstrap values. Similar results were obtained using Neighbor joining (NJ) phylogenetic analyses (data not shown). Class I is represented by species from monocots, eudicots, and gymnosperms. Class II and Class III are represented by only sequences from angiosperms. The subdivision of Class I in two subclades is the result of a duplication event that happened in the ancestor of gymnosperms. The only known basal angiosperm (*Saruma henryi*) CAD (*SheCAD_A*) [38] is located in Class II. Class I contains the two *Arabidopsis *(*AtCAD5 *and *AtCAD4*) [32] CAD genes previously shown to be associated with lignin biosynthesis. It also includes *PoptrCAD4 *which we found to be preferentially expressed in xylem (this study). All the other genes from *Populus trichocarpa *and *Arabidopsis *were distributed in Class II and Class III. Clustering of several genes from monocots, eudicots, and gymnosperms suggest within-species duplications.

### Histochemistry of lignin deposition in *P. trichocarpa *tissues

**Figure 4:**
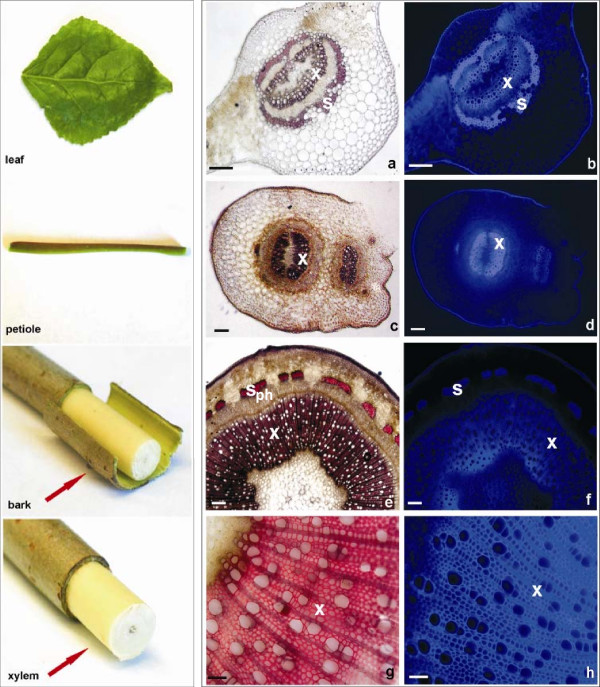
**Lignification pattern in *Populus *tissues selected for qRT-PCR studies**. Far left column displays organs and tissues used (Leaf blade, Petiole, bark, Xylem). Middle column shows lignin deposition, visualized under the light microscope after phloroglucine-HCl staining (red color). Right column shows lignin distribution by fluorescent microscopy (autofluorescence). a, b – cross section of leaf vascular bundle, c, d – petiole cross section, e, f – transverse section of stem segment, g, h – secondary xylem from stem. Abbreviations: x – xylem, ph – phloem, s – schlerenchyma. Bars = 100 μm.

Before analyzing the expression of CAD genes using Real time RT-PCR, we analyzed lignin deposition patterns in the tissues of plants by staining with phloroglucinol and observation by light and fluorescent microscopy. The lignin distribution pattern under UV light was similar to that of staining with acidified phloroglucinol, indicating that the same tissues were lignified. In leaf tissues lignin was detected mainly in the xylem of vascular bundles and in schlerenchyma fibers surrounding vascular tissues (Fig. 4a, b). Petioles were lignified only in secondary cell walls of xylem and in the extensive hypodermal band of schlerenchyma (Fig. 4c, d). The most heavily lignified tissues were observed in stem segments. The bark of the stem, including phloem sieve tube cells, and parenchyma were not lignified (Fig. 4e). In the bark, lignin was detected only in schlerenchyma fibers at the outer part of phloem (Fig. 4e, f). Secondary xylem with thickened secondary cell walls showed the strongest reaction, demonstrating large amounts of lignin distributed in the tracheary vessels and fibers (Fig. 4g, h).

### Expression analysis of *Populus *CAD genes

**Figure 5:**
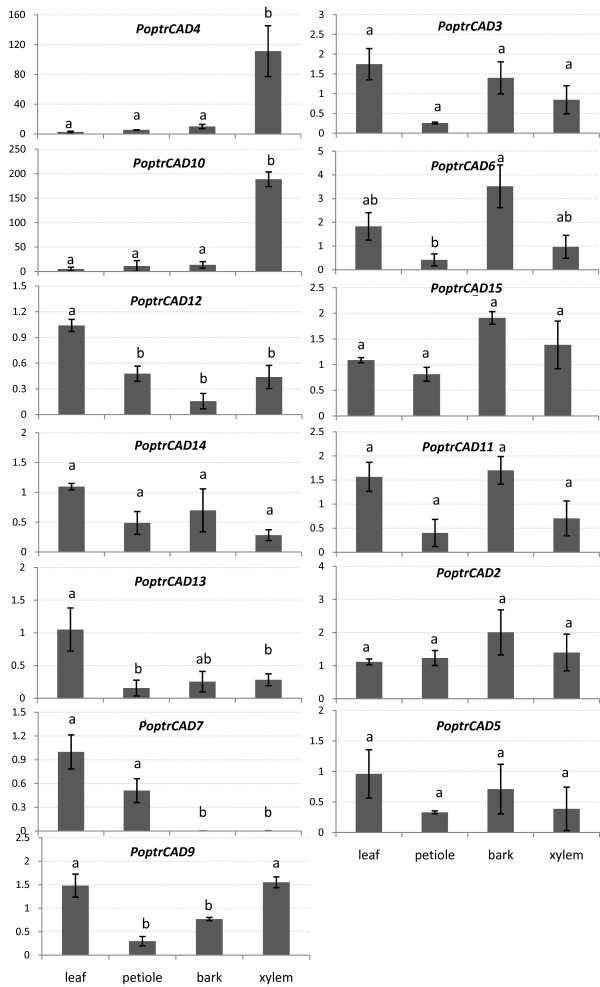
**Quantitative expression of *Populus *CAD genes**. The name of each gene is indicated at the top of each histogram. Tissues studied are shown at the bottom of the diagrams. Means designated by the same letter do not differ significantly according to Tukey's HSD test; P < 0.05).

Of the 15 CAD genes found in *Populus*, we analyzed the expression of 13 (see Additional file 1) in several different tissues that were selected based on the previous histochemical studies (Fig. 4). Expression analysis using quantitative real-time RT-PCR (Fig. 5) showed that all CAD genes are expressed in leaves, petioles, bark and xylem, but at different levels among the tissues. *PoptrCAD7*, for example, is expressed in leaves and petioles, but presents a very low expression in the bark and xylem. The expression patterns vary widely between genes, which were sorted into four groups based on the expression profiles observed in different tissues (Fig. 3). Group 1 (*PoptrCAD4*; *PoptrCAD10*) is represented by genes strongly expressed in xylem (lignin associated) – 100 times more highly expressed in xylem than the other CAD genes. Statistical analysis using the Ward linkage method showed that group 1 is significantly different in expression from the other three groups. One-way ANOVA analysis showed that the expression of *PoptrCAD4 *and *PoptrCAD10 *(group 1) in the xylem was statistically different from each other (p < 0.005) with *PoptrCAD10 *more expressed. Group 2 (*PoptrCAD13*, *PoptrCAD7*, *PoptrCAD12*) genes are expressed in all tissues but are most highly expressed in leaves. The group 3 (*PoptrCAD9*) gene is preferentially expressed in leaves and xylem. Genes from group 4 (*PoptrCAD2, PoptrCAD3, PoptrCAD5, PoptrCAD6, PoptrCAD11, PoptrCAD14, PoptrCAD15*) did not show any significant expression differences between tissues. As indicated in Fig. 3, group 1 genes are distributed in Class I and Class II, group 2 and group 4 genes are distributed in Class II and Class III, while gene from group 3 belong to Class II.

Analysis of gene duplicates in *Populus *showed that *PoptrCAD2 *and *PoptrCAD11 *presented similar expression patterns in that they both did not show any significant expression differences between tissues. Similarly, *PoptrCAD3 *and *PoptrCAD5 *presented similar expression profiles in the tissues analyzed.

## Discussion

### Organization of CAD genes in *Populus*

Previous studies reported the identification of complete sets of CAD genes from the model plant species *Arabidopsis *and *Oryza *[29,30], along with several sequences from non-model species [29,30,36]. Those studies [29,30,35] reported also preliminary phylogenetic trees for CAD genes based on a limited set of sequences mainly from *Arabidopsis*, *Populus*, and *Oryza *lineages. Moreover, no phylogenetic study including genome organization, gene structure, phylogeny, and expression profiling has been reported to date on the model tree species *Populus*. Here, we report the analysis of the phylogeny of CAD genes using five complete genome sequences and a set of genes from various land plant lineages. We also analyzed the structure of CAD genes and their promoters as well as their physical organization on *Populus *chromosomes and their expression patterns in various plant tissues.

Our study of the organization of CAD genes showed that chromosome duplications contributed significantly to the duplication of CAD genes in the *Populus *genome. Similar results were reported for *Arabidopsis *and *Oryza *[30,31]. Almost 80% of genes in *Arabidopsis *and *Populus *were distributed on duplicated regions. We cannot be sure if those duplications happened independently in both species or if some of them have occurred in the ancestor of those species. The distribution of several *Populus *duplicates on segmental duplications reported previously [35,39] associated with the Salicoid duplication event that occurred 65 million years (myrs) ago indicates that most CAD gene duplications happened in the ancestor of *Populus*. Dating duplications in *Populus *using a rate of 1.5 × 10^-8 ^synonymous substitutions per synonymous site per year as proposed by Koch et al., (2000) showed that most of them have occurred between 4 and 15 myrs ago. At least three other duplication events may have occurred prior to the large duplication event at ~20, ~30, and ~38 myrs ago. This timing corresponds to the large duplication event reported previously (~13 myrs) [35,40] that occurred in the ancestor of *Populus*. However, based on the molecular clock timing, all duplication events seem to be postdating the earliest fossils of *Populus*, which are dated at ~58-myr ago (Eckenwalder, 1996). The comparative timing of the duplication event reported in previous work [40] and in this study suggest that the timing of *Populus *duplications is not accurate as the *Populus *genome is evolving slowly compared to *Arabidopsis*. Nevertheless, the distribution of *Populus CAD *genes on segmental duplications associated with the Salicoid duplication, the agreement between our duplication timing result and those reported previously (Streck et al., 2005), and the distribution of CAD genes on the phylogenetic tree suggest that most of those duplications happened in the ancestor of *Salicaceae*. The retention of duplicate genes in the *Populus *genome is not surprising as the genome of this species has been suggested to evolve at a slow rate compared to *Arabidopsis*[35]. However, this retention seems to be common to several species such as *Arabidopsis *[36], *Oryza *[30,36], *Populus *(this tudy), *Vitis *(this study), and *Medicago *(this study). Whether the duplicated CAD genes correspond to genetic redundancy or have evolved divergent functions, they must be involved in important processes in the plant to be retained in these two very different eudicot species. In sharp contrast, only one rice CAD gene was found on a large duplicated block We are not sure if *Oryza *CAD genes did not experience large duplications or if most of the duplicates have already been lost. It is noteworthy that four *Oryza *CAD genes located at the same locus evidently evolved by inverted duplications. This may represent an alternative mechanism of CAD gene family evolution in rice versus Eurosids.

Three patterns of intron-exon structure were observed among CAD genes. Patterns 1 and 2 are characterized by 5 exons and 4 introns, while Pattern 3 CAD genes have 6 exons and 5 introns. Pattern 1 was detected in eudicots (*Arabidopsis*, *Populus*) and monocots (rice), while pattern 2 was found in eudicots (*Arabidopsis *and *Populus*) and a basal angiosperm, i.e *Liriodendron tulipifera *(Haiying Liang, personal communication). Pattern 3 was detected in eudicots and monocots (this study) as well as in gymnosperms [41]. Pattern 2 was found in several *bona fide *CAD genes (Class I) as well as some genes from Class II. Based on these results, at least pattern 2 and pattern 3 existed in the ancestor of angiosperms. This is confirmed by the dating of the duplication events of *Populus *genes, as the duplications that generated genes with pattern 1 were recent compared to the one that generated genes with pattern 2 and pattern 3. Furthermore, *Oryza *seems to have several other specific variant patterns of introns/exons that may have evolved in rice or the ancestor of the *Poaceae*, some lacking introns which were apparently generated by transposable elements. This diversification in rice could be linked to the high evolution rate of *Poaceae *genes compared to the two eudicot model species.

### CAD gene family is divided into three main classes

Phylogenetic analyses showed that CAD genes are divided into three classes based on their AA and nt sequences. CAD class I included sequences from monocots, eudicots, and gymnosperms clades. Class II and Class III include sequences from monocots and eudicots. This indicates that the evolution of Class II and Class III happened in the ancestor of angiosperms, or at least prior to the split of monocots and dicots. This result is similar to the one published recently by Tuskan and collaborators [35] using mainly sequences from monocots and eudicots. The tree obtained in this study differs from previous analyses [29,35] which grouped the CAD genes in *Arabidopsis *into three classes, with the gymnosperm sequences clustering in a separate class [29]. It is also different from the tree published previously [30] showing a distribution of CAD genes in two mains classes. The difference between our phylogeny and the ones published previously [29,30,35] could be due to the inclusion of a broader set of species in this study. Several sequences from various species cluster close to each other; suggesting that there are species- or lineage-specific CAD gene duplications. This is in accordance with the distribution of ~80% of CAD genes from *Arabidopsis *and *Populus *on duplicated blocks, some of which may have been generated by lineage-specific duplications. It is noteworthy that except for the *bona fide *genes (*AtCAD4 *and *AtCAD5*) which belong to Class I, all the other *Arabidopsis *CAD genes (previously known as "CAD-like genes") fell into Class II and Class III in our analysis. Other known *bona fide *CAD genes which were grouped into Class I in our study included *Populus tremuloides PtrCAD_B *(*PtCAD*) (Li et al., 2001), *Oryza OsCAD2 *(*OsCAD2*) (Tobias et al., 2005), and *Eucalyptus Egu_A *(*EuCAD2 *or *EgCAD*) (Grima-Pettennati et al., 1993). *Populus tremuloides *SAD gene (Li et al., 2001) and *Arabidopsis *genes (*AtCAD4 *and *AtCAD5*) [33], which were reported as being involved in lignin biosynthesis were located in class II in our study. *PoptrCAD4 *and *PoptrCAD10*, which were highly preferentially expressed in xylem, were found in Class I and Class II respectively. Based on the close distribution of *PoptrCAD10 *to *Populus tremuloides *SAD on the phylogenetic tree; it seems that *PoptrCAD10 *is the ortholog of *Populus tremuloides *SAD gene. This result confirms previous results (Li et al., 2001) showing that there are two genes (CAD and SAD) involved in lignin biosynthesis in xylem from *Populus trichocarpa *and *Populus tremuloides*. Class III is represented by *ATCAD1 *which was reported presenting similar expression profile as *bona fide *genes (*AtCAD4 *and *AtCAD5*) in *Arabidopsis *plant tissues [33] even though their CAD catalytic activity could not be proven.

Previous studies reported the distribution of CAD genes in several classes and suggest that with the exception of *bona fide *lignin biosynthesis genes, all others are involved in plant defense (Tuskan et al., 2006). The distribution of most *bona fide *CAD genes from various species in Class I in this study favors such a functional distinction between Class I and II genes. However, the exceptions of *PoptrCAD10 *(SAD) from *Populus trichocarpa*, *PtrCAD1 *(SAD) from *Populus tremuloides*, and *AtCAD8 *and *AtCAD7 *[33], which were reported as being lignin associated and are distributed in class II, rule against this hypothesis. The most probable hypothesis is that some genes from class II evolved a modified expression profile or function such as plant defense against pathogens. The gain of function hypothesis for the genes from Class II is supported by the fact that some genes from this class are still associated with lignin biosynthesis in xylem. Two alternate hypotheses could explain the evolution of defense function of CAD genes. The first hypothesis is that CAD genes evolved defense function after the split of Class II and Class III from Class I genes. The second hypothesis is that the functional divergence of CAD genes occurred before the split of Class II and Class III from Class I. Further functional analysis of genes from Class I and Class II will be needed to answer this question.

### CAD genes show different expression profiles in various *Populus *tissues and possibly divergent functions

The high rate of duplication of CAD genes and the retention of most duplicates raises the question of their functional redundancy. Quantitative expression analysis showed that among the CAD genes studied, four expression patterns were presented in the tissues studied. *PoptrCAD4 *and *PoptrCAD10 *from expression-group 1 were differentially expressed in xylem tissues and are associated with lignin biosynthesis. This conclusion is supported by the distribution of *PoptrCAD4 *into Class I with several previously reported *bona fide *CAD genes [33]. *PoptrCAD10 *clusters in Class II closely with the *Populus tremuloides *SAD gene and *Arabidopsis AtCAD8 *and *AtCAD7*, which has been reported as being involved in lignin biosynthesis [14,33]. Promoter analysis (Table 1) showed that *PoptrCAD4 *possess several motifs involved in stress response such as defense/stress responsiveness, MeJA, ABA, and light responsiveness. In contrast, *PoptrCAD10 *possess motifs involved in the interaction with zinc finger binding transcription factor and in the response to auxin. This result suggests that while both genes are involved in lignin biosynthesis, *PoptrCAD4 *expression may be modulated under biotic stress conditions. Genes from expression-groups 2 and 3, which are preferentially expressed in leaves could correspond to a defense-related lignin- biosynthesis pathway or other defense pathway as suggested previously [42,43]. They possess motifs involved in response to herbivory, wound, and MeJA. MeJA plays a key role in plant defense against various biotic and abiotic stresses [44,45]. Preliminary expression profiling of these genes in *Populus *under stress conditions confirmed this hypothesis as some of those genes increase their expression under herbivore (Gypsy moth) stress (data not shown). This result is not surprising as most pathogen invasions occur in the leaves. It is also in accordance with previous studies showing that *CAD-like *genes are involved in plant defense [43]. CAD genes from expression-group 4, which did not present any expression difference between various plant tissues, possess several motifs that are involved in response to MeJA, wound, fungal elicitor, stress and defense responsiveness, and ethylene. Those genes may function in lignin biosynthesis under other stress conditions. Comparison of gain/loss of motifs in the promoter region did not allow the identification of probable motifs underlying the difference in expression profiles between *bona fide *CAD genes and the *CAD-like *genes.

From a functional perspective, the lingering question is why diverse copies of CAD genes from Class II and Class III have been maintained within plant genomes. One can ask if CAD genes from Class II and Class III, except *PoptrCAD10*, are involved only in plant defense or some of them can still compensate the function of bona *fide *CAD genes (*PoptrCAD4 *and *PoptrCAD10*) in lignin biosynthesis in xylem. The expression profile differences between CAD-like genes from Class II and Class III in the various tissues analyzed, added to the differential distribution of several motifs involved in various developmental and physiological processes in their promoter regions suggests that there is a functional specialization of CAD genes in various tissues and under various development and stress conditions. Expression analysis of two pairs of paralogs (*PoptrCAD3 *and *PoptrCAD5*, *PoptrCAD2 *and *PoptrCAD11*) showed that they have similar expression profiles. This suggests that the duplication of those genes did not result in divergence of their expression profile and function. However, we cannot rule out this hypothesis as these duplicate genes present different motifs for responses to various stresses in their promoter regions. Moreover, the expression of those duplicate genes could be regulated at the protein level. Therefore, the quantification of protein corresponding to those genes is needed to confirm this hypothesis.

## Conclusion

In conclusion, we identified 15 CAD genes in *Populus *and found that most of them were located in the genome on duplicated blocks. We demonstrated that CAD genes in land plants were distributed in three phylogenetic classes of which two may have originated from duplications in the ancestry of all angiosperms. Class I genes function in lignin biosynthesis in xylem while genes from Classes II and III may function under stresses conditions. Promoter sequence analysis and preliminary results on expression profiling of CAD genes in tissues suggest CAD genes have evolved divergent expression profiles or functions.

## Methods

### CAD sequences used in phylogenetic analysis

CAD sequences used in phylogenetic analyses (see Additional file 1) include sequences generated in this study as well as sequences retrieved from different databases. We used sequences from plants with fully sequenced genomes as well as other taxons representing key positions on the angiosperm phylogenetic tree. CAD sequences from *Arabidopsis*, *Oryza*, and *Populus *were retrieved from TAIR , TIGR , and the Joint Genome Institute . CAD sequences from the newly sequenced genomes of *Carica papaya*, *Vitis vinifera*, and *Medicago truncatula *were retrieved from The Hawaii Papaya Genome Project [46], the *Vitis *genome [47], and the Medicago Sequencing Resources , respectively. CAD sequences from various non model species were retrieved from TIGR Plant Genomics databases , GeneBank  TIGR , and the floral genome project database [38] databases. Sequences were carefully inspected and corrected for annotation errors before use.

### Intron-exon structure and promoter analysis of CAD genes

The exon/inron structure of CAD genes was retrieved from the Joint Genome Institute  web site. For genes for which complementary DNA (cDNA) sequences were available, the structure is checked by aligning genomic and cDNA sequences. Promoter analysis was done by querying all CAD genes against TRANSFAC [48] and PlantCARE [49].

### CAD sequences alignment and phylogenetic analyses

CAD nucleotide cDNA sequences were translated into protein sequences. The inferred protein sequences were then aligned using Muscle with default parameters [50], and manually adjusted. Phylogenetic analyses were performed on the aligned amino acid (AA) sequences, as well as on the nucleotide sequences that were aligned to match the AAa. The GTR model [51] was found to be optimal for nt datasets, assuming among site rate heterogeneity and a proportion of invariable sites (GTR+G+I), while the WAG model [52], assuming among site rate heterogeneity (WAG+G), was found to be the best fit for the aa sequences. These models were used for Maximum Likelihood (ML) analyses implemented in PHYML v. 2.4.4 [53]. 250 bootstrap replicates were run to estimate branch support. Neighbor-joining (NJ) analyses were performed in MEGA4. Since the models of best fit were not available here, we chose the JTT and Tajima-Nei models, using pairwise deletion and assuming gamma distributed site rates. 500 bootstrap replicates were run to estimate branch support.

### Histochemistry of lignin deposition analyses

For visualization of lignin distribution, plant material (leaf blades, petioles, and stem) was free-hand sectioned with a razor blade. Sections were stained with phloroglucinol (2% w/v phloroglucinol acidified in 6 M HCl), mounted in glycerol and observed under an Olympus BX51 light and fluorescent microscope, equiped with a SPOT II RT digital camera.

### RNA isolation and cDNA synthesis

Leaves, petioles, stem secondary cortex and stem xylem were collected from young hybrid *Populus *OGY (*P. deltoides *× *P. nigra*) young trees grown in a culture chamber at 25°C and 18°C in the day and night, respectively. The plants were grown at 16 h/8 h day/night regime and at 60% humidity. Tissues were harvested and immediately frozen in liquid nitrogen and stored at -80°C until used for RNA isolation. Total RNA was isolated using CTAB method [54] with minor modifications. The RNA quality and concentration was assessed using an Agilent 2100 Bioanalyzer (Agilent Technologies). To remove any contaminating genomic DNA, RNA samples were treated with RNAse free DNAse (Applied Biosystems) before real time RT-PCR experiments. RNA was reverse transcribed using random primers from the High Capacity cDNA Reverse Transcription kit (Applied Biosystems) and random primers following the manufacturer's recommendations. One microgram of total RNA from each sample was reverse-transcribed to generate cDNA.

### CAD expression analysis using quantitative real time RT-PCR

Quantitative real time PCR reactions were prepared using the SYBR Green Master Mix kit (Applied Biosystems) and performed in an Applied Biosystems 7500 Fast Real-Time PCR system (Applied Biosystems) with default parameters. Primers used in this study (see Additional file 3) were designed using Primer Express^® ^software (Applied Biosystems) or primer 3 software (The Whitehead Institute for Biomedical Research, Cambridge, MD, USA). We used the gene encoding the 18S rRNA as an endogenous control to normalize for template quantity. The real-time PCR protocol was performed as following: denaturation by a hot start at 95°C for 10 min, followed by 40 cycles of a two-step program (denaturation at 95°C for 15 sec and annealing/extension at 60°C for 1 min). Dissociation curves were used to verify the specificity of PCR amplification. For each tissue, samples from three different trees were used. Triplicate experiments were analyzed for each tissue and each tree. Data was evaluated using the 7500 Fast System SDS software procedures (Applied Biosystems). Statistical analyses were performed using Statistica 6.0 software (StatSoft Poland Inc., Tulsa, OH, USA).

## Abbreviations

CAD: Cinnamyl alcohol dehydrogenase; nt: nucleotide; AA: amino acids; cDNA: complementary DNA; RT-PCR: Reverse transcriptase polymerase chain reaction; PAL: phenylalanine ammonia-lyase; HCT: hydroxycinnamoyl:CoA shikimate/quinate hydroxycinnamoyl transferase; 4CL: 4-coumarate:CoA ligase; CCR : cinnamoyl-CoA reductase; C3H: p-coumarate 3-hydrolase.

## Authors' contributions

AB retrieved, curated, annotated, and aligned the CAD nucleotide and protein sequences. He analyzed the gene structure, ran the phylogenetic analyses, supervised ABZ, AC, UP, SD, and PY, and wrote the manuscript. ABZ contributed to the RNA preparation and the expression analyses. UP and AC contributed to curating and aligning the CAD sequences. SD collected *Populus *tissues and participated in RNA preparation. PY contributed to promoter sequence analysis. This project was initiated by AB and JC. JC directs The Schatz Center for Tree Molecular Genetics at Penn State which funded the project, and he contributed to the evaluation and discussion of the results, and assisted in the preparation of the manuscript. All authors read and approved the final manuscript.

## Supplementary Material

Additional file 1 **List of plant genes used in CAD gene phylogenetic analyses**. The gene names used in this study, the accession number, species, the database source, and names of previously published genes are indicated. Click here for file

Additional file 2 **List and description of nucleotide motifs discovered in the promoter regions of *Populus *CAD genes**. Click here for file

Additional file 3 **List of *Populus trichocarpa *primers used for expression profiling of CAD genes in hybrid Populus (*P. deltoides *× *P. nigra*).** Click here for file
